# Platycodin D2 enhances P21/CyclinA2-mediated senescence of HCC cells by regulating NIX-induced mitophagy

**DOI:** 10.1186/s12935-024-03263-y

**Published:** 2024-02-19

**Authors:** Lili Sun, Yaru Li, Renshuang Zhao, Qinlei Fan, Fei Liu, Yilong Zhu, Jicheng Han, Yunyun Liu, Ningyi Jin, Xiao Li, Yiquan Li

**Affiliations:** 1 Medical College, Yanbian University, Yanji, 133002 People’s Republic of China; 2 Department of Head and Neck Surgery, Tumor Hospital of Jilin Province, Changchun, 130000 People’s Republic of China; 3 Academician Workstation of Jilin Province, Changchun University of Chinese Medicine, Changchun, 130117 People’s Republic of China; 4 Chinese Center for Animal Hygiene and Epidemiology, Qingdao, 266032 People’s Republic of China; 5 Changchun Veterinary Research Institute, Chinese Academy of Agricultural Sciences, Changchun, 130122 People’s Republic of China; 6 Jiangsu Co-Innovation Center for Prevention and Control of Important Animal Infectious Diseases and Zoonoses, Yangzhou University, Yangzhou, 225009 People’s Republic of China

**Keywords:** Mitophagy, Cell senescence, Platycodin D2, Hepatocellular carcinoma

## Abstract

**Background:**

Hepatocellular carcinoma (HCC) cells usually show strong resistance to chemotherapy, which not only reduces the efficacy of chemotherapy but also increases the side effects. Regulation of autophagy plays an important role in tumor treatment. Cell senescence is also an important anti-cancer mechanism, which has become an important target for tumor treatment. Therefore, it is of great clinical significance to find anti-HCC drugs that act through this new mechanism. Platycodin D2 (PD2) is a new saponin compound extracted from the traditional Chinese medicine Platycodon grandiflorum.

**Purpose:**

Our study aimed to explore the effects of PD2 on HCC and identify the underlying mechanisms.

**Methods:**

First, the CCK8 assay was used to detect the inhibitory effect of PD2 on HCC cells. Then, different pathways of programmed cell death and cell cycle regulators were measured. In addition, we assessed the effects of PD2 on the autophagy and senescence of HCC cells by flow cytometry, immunofluorescence staining, and Western blotting. Finally, we studied the in vivo effect of PD2 on HCC cells by using a mouse tumor-bearing model.

**Results:**

Studies have shown that PD2 has a good anti-tumor effect, but the specific molecular mechanism has not been clarified. In this study, we found that PD2 has no obvious toxic effect on normal hepatocytes, but it can significantly inhibit the proliferation of HCC cells, induce mitochondrial dysfunction, enhance autophagy and cell senescence, upregulate NIX and P21, and downregulate CyclinA2. Gene silencing and overexpression indicated that PD2 induced mitophagy in HCC cells through NIX, thereby activating the P21/CyclinA2 pathway and promoting cell senescence.

**Conclusions:**

These results indicate that PD2 induces HCC cell death through autophagy and aging. Our findings provide a new strategy for treating HCC.

**Graphical Abstract:**

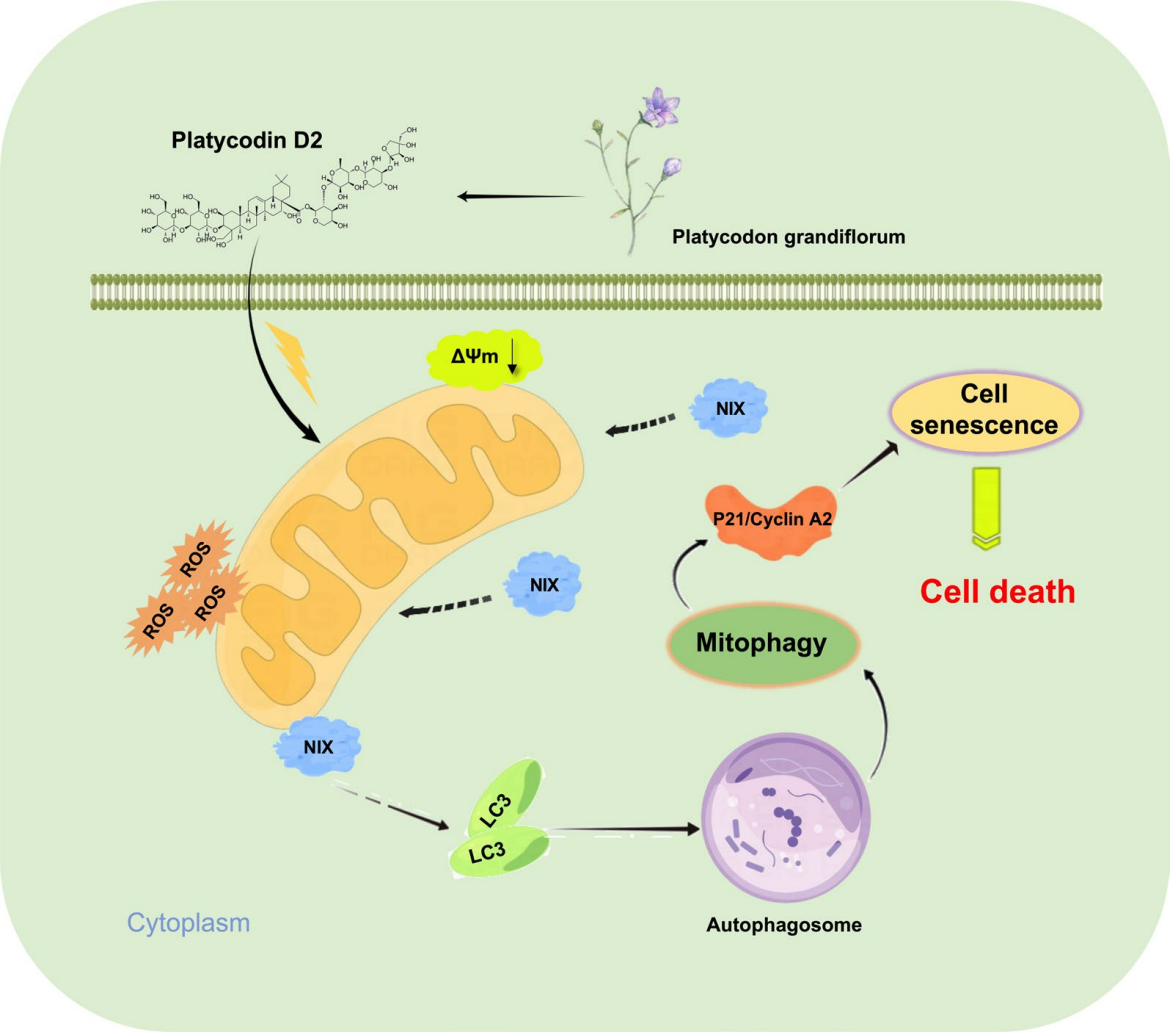

**Supplementary Information:**

The online version contains supplementary material available at 10.1186/s12935-024-03263-y.

## Introduction

Hepatocellular carcinoma (HCC) is one of the most common malignant tumors, accounting for approximately 85% of all cases of primary liver cancer. Therefore, "Liver cancer" is commonly referred to as ‘‘HCC’’ [1, 2]. Surgery is the first treatment choice for early-stage liver cancer, while chemotherapy is commonly used for treating advanced and metastatic HCC. However, HCC cells are usually resistant to chemotherapy, and the response rate to chemotherapy is only about 10%-20%, which shortens the overall survival of patients [3]. Acquired resistance to chemotherapy is a sign of HCC progression [4]. In addition to routine treatment, interventional therapy, targeted therapy, and immunotherapy have been used in recent years. However, these modalities of cancer treatment have their own limitations [5]. For example, the response to interventional therapy depends on several factors, such as the size and location of the tumor, and there is a high chance of recurrence and metastasis after treatment. Similarly, the efficacy of targeted therapy relies on the heterogeneity and immune tolerance of cancer cells. Immunotherapy modalities, including CTLA-4 inhibitors, PD-1/PD-L1 inhibitors, and CAR-T cell therapy, have attracted much attention in recent years [6]. However, immune escape and tolerance of HCC cells can undermine the efficacy of immunotherapy. Recent clinical and preclinical studies have shown that available drugs cannot effectively treat HCC. Therefore, new drugs or mechanisms are needed to improve the treatment of HCC.

Inducing cell senescence can inhibit cell proliferation. Senescent cells generally activate senescence-associated β-galactosidase and other key proteins, such as P16, P53, and P21, that mediate cell cycle arrest [7]. P21 and P16 control the cell cycle and induce cell death or permanent cell cycle arrest when abnormally expressed.

Cell senescence also induces the secretion of various chemokines, cytokines, and proteases, which in turn constitute the senescence-associated secretory phenotype (SASP). Accumulating evidence shows that promoting cellular senescence is an effective anti-tumor mechanism [8]. In addition, pro-aging approaches can synergistically improve the efficacy of immune checkpoint inhibition because SASP can summon various immune cells [9]. Moreover, induced senescence has been shown to enhance drug delivery and T cell infiltration [10], which in turn can reduce cancer cell resistance to chemotherapy. Therefore, it is of great importance to discover or design effective drugs for promoting tumor cell senescence.

Mitophagy is a conserved catabolic process that plays an important role in maintaining cellular homeostasis through the selective removal of dysfunctional mitochondria. Previous studies found that Pink1 translocates Parkin from the cytoplasm to the surface of depolarized mitochondria, thereby promoting mitochondrial protein ubiquitination and inducing mitophagy [11]. Studies have shown that NIX/BNIP3L, FUNDC1, and BNIP3 also play important roles in mitophagy [12]. Among them, NIX is a dual-nature protein, which can not only induce apoptosis but also induce autophagy [13].

NIX-mediated mitophagy has been implicated in many human diseases, including neurological metabolic diseases and cancer. Among them, the role of NIX in cancer cells is still controversial. Initially, NIX was considered to be a tumor suppressor gene. However, recent studies have found that NIX-mediated autophagy can cause tumor cell death. NIX has been reported to induce autophagy by recruiting TR3 to mitochondria, thereby inducing melanoma cell death [14]. Furthermore, studies in glioma cells have shown that NIX is involved in mitophagy-mediated cell death induced by the natural compound AT 101 [15]. These studies all suggest that NIX-mediated mitophagy accelerates cancer cell death. Therefore, it is of great interest to develop a drug that promotes mitophagy.

Some studies have shown that autophagy is associated with cellular senescence [16]. Initially, autophagy was deemed to prevent cellular senescence; however, the opposite conclusion has been reported by some recent studies. Specifically, they claimed that autophagy can promote cellular senescence by promoting the synthesis of senescence-related secreted proteins [17, 18]. Other Studies have shown that excessive autophagy impairs organelle quality control and energy homeostasis, thereby leading to cellular senescence [19]. Other studies have found that sustained p38α activation can increase the number of autophagosomes and enhance autophagic flux, thereby inducing cell senescence [20]. Both autophagy and senescence have anti-tumor effects. Autophagy can also promote cellular senescence, suggesting that activation of autophagy and cellular senescence is a potential cancer treatment approach.

The traditional Chinese medicine *Platycodon grandiflorum (Jacq.) A. DC.* belongs to the family Platycodon grandiflorum and its main medicinal derivative is the dried rhizome. Recent studies on the active derivatives of Platycodon grandiflorum showed that platycodon grandiflorum contains various medicinal constituents, including saponins, polysaccharides, flavonoids, polyphenols, and polyacetylene. Notably, the triterpene saponin isolated from platycodon grandiflorum, namely Platycodin, was reported to be the main bioactive component. Currently, Platycodin D (PD) is the most studied active derivative from the Platycodin family. As a main medicinal derivative of Platycodon grandiflorum, PD has a significant anti-tumor effect. Studies have shown that PD has cytotoxic effects on various cancer cells, including liver cancer cells. However, PD is also cytotoxic to normal cells [21–23]. Platycodin D2 (PD2), another novel saponin extracted from Platycodon grandiflorum, has shown anti-inflammatory, anti-hepatitis B virus, anti-hepatitis C virus, and anti-tumor properties [24–26]. However, it was effective against some types of cancer cells, including A549, SKOV-3, SK-MEL-2, XF498, and HCT15 tumor cells, and the specific mechanism is not clear [27–29].

Currently, there are few studies on the anti-tumor property of PD2, and the underlying molecular mechanisms have not been clarified. This study aimed to measure the effect of PD2 on autophagy and cellular senescence of HCC cells. We conducted in *vitro* and in *vivo* studies to investigate the effect of PD2 on the autophagy and senescence of HCC cells, providing a theoretical basis for the future development of anti-tumor drugs based on PD2.

## Materials and methods

### Cells and reagents

The liver cancer cell lines, Huh-7, MHCC97H, HCCLM3, HepG-2, and SK-Hep1, Huh-6, and normal liver cell lines, THLE-2 and L02, were purchased from the Guangzhou Saiku Biotechnology Co. (Guangzhou, China). PD2 (cat. HY-N4087), 3-methyladenine (3-MA) (cat. HY-19312), chloroquine (CQ) (cat. HY-17589A), and rapamycin (cat. HY-10219) were purchased from MedChemExpress (MCE, USA).

We purchased the control siRNA, NIX, and P21 siRNAs from RiboBio (China). Si-NIX (cat. SIGS0005709-1) and si-P21 (cat. SIGS0001992-1) were used in this study. We transfected 30 nM siRNA into cells using the transfection reagent in the kit.

Human cyclin A2 cDNA was purchased from the Public Protein/Plasmid Library, (Jiang su, China) and cloned into the pCDNA 3.1 plasmid. Following the manufacturer’s protocol, the cyclin A2 plasmid and corresponding empty vector were transfected into HCC cells using Advanced DNA/RNA Transfection Reagent (ZETA life, USA).

Five- to six-week-old female BALB/c nude mice were used in this study. Mice were fed in a specific pathogen-free environment at the Animal Experimental Center of the Changchun University of Chinese Medicine. Their food was sterilized with cobalt-60 radiation, and their ultrapure water was sterilized by autoclaving. This study was approved by the Institutional Animal Care and Use Committee of Changchun University of Chinese Medicine (Approval No. 2022062).

### Cell viability assay

HCC cells were cultured in DMEM medium supplemented with 10% FBS and 1% penicillin/streptomycin dual antibody and incubated in an incubator at 37 ℃ and 5% CO_2_. HCC cells were passaged into 96-well plates at a rate of 5 × 10^4^/well. After 24 h, Huh-7, MHCC97H, HCCLM3, HepG-2, SK-Hep1, and Huh-6 cells and normal liver cells, THLE-2 and L02, were treated with different doses of PD2 from 1 to 100 μM. 5-FU was used as a positive control. After 48 h, the culture medium was replaced with 100 μL of medium containing 10 μL of the CCK-8 reagent (Cat#C0038, Beyotime Biotechnology). After 2 h of incubation at 37℃ and 5% CO_2_, the absorbance value was measured at 450 nm.

### Detection of apoptosis rate

HCC cells were passaged into six-well plates (2 × 10^5^/well) and cultured in an incubator for 24 h. Double-free DMEM was added, and then 10 μM of PD2 was added to each well. After 48 h of incubation, the culture medium was removed and washed with PBS. Cells were digested with trypsin and centrifuged at 500 g for 5 min. The cell precipitate was washed with PB and resuspended in 1 × binding buffer. Subsequently, 5 μL of FITC and 5 μL of PI were added. After gentle blowing, cells were stained in the dark at room temperature for 20 min, and analyzed using a Beckman Coulter flow cytometer (CytoFLEX A00-1–1102, USA).

### Cell cycle detection

HCC cells were seeded in six-well plates at a density of 2 × 10^5^ cells/well. After 24 h of culture in the incubator, double-free DMEM was added. Thereafter, 10 μM of PD2 was added. After 48 h of culture in the incubator, PBS or the drug was removed, and PBS was washed. Cells were digested with trypsin, centrifuged for 5 min (500 g), and washed with PBS. Precooled 70% ethanol was added to the samples, and the samples were placed in a refrigerator at 4 °C for 2 h. Then, the samples were centrifuged for 5 min (300 g), and PBS was added to re-suspend the cell precipitate. After centrifugation at 300 g for 5 min, the supernatant was removed, and PBS (0.5 mL) was added. Then, 5 μL of cell cycle solution was added, and the mixture was fully shaken and mixed with a vortex oscillator in the dark. The mixture was placed in an incubator for 15 min and analyzed using a Beckman Coulter flow cytometer (CytoFLEX A00-1-1102, USA).

### Autophagy change detection

Cells were grown in six-well plates and treated with PD2 for 24 h. Next, cells were transfected with the plasmid EGFP-LC3. After 48 h, cells were fixed with 4% paraformaldehyde for 15 min, and changes in the green fluorescence of LC3 were observed under a confocal microscope (CARL ZEISS LSM980, Germany).

The cells were grown in six-well plates and treated with PD2 for 24 h. Next, the cells were infected with mRFP-GFP-LC3 double labeled adenovirus (Cat#HB-AP2100001, Hanbio Biotechnology, China) at a dose of 20 MOI. After 48 h, the cells were fixed with 4% paraformaldehyde for 15 min, and changes in the green fluorescence of LC3 were observed under a confocal microscope (CARL ZEISS LSM980, Germany).

The cells were grown in six-well plates and treated with PD2 for 48 h. The cells were collected and centrifuged, and then subjected to fixation, dehydration, embedding, slicing, and staining. Autolysosome formation was observed using a transmission electron microscope (JEOL 1400, Japan).

### Detection of mitochondrial membrane potential

The qualitative and quantitative changes in mitochondrial membrane potential can be detected using JC-1 staining. HCC cells were passaged into 12-well plates at 2 × 10^5^/well. After 24 h, 10 μM PD2 was added to each well. After 48 h, the cells were stained with JC-1 staining solution. After 15 min of staining, cells were washed three times and then observed using a fluorescence microscope.

HCC cells were passaged into 96-well plates at 5 × 10^4^/well and treated with 10 μM PD2 24 h later. After 48 h, the culture medium was discarded and replaced with 100 μL of JC-1 staining solution. Absorbance was measured at 435 and 585 nm after incubation at 37 ℃ for 2 h.

The changes in mitochondrial membrane potential were measured by TMRM staining. Hepatoma cells were passaged into 12-well plates at 2 × 10^5^/well. After 24 h, 10 μM of PD2 was added to HCC cells. After 48 h, the cells were stained with TMRM staining solution, washed three times after 30 min of staining, and then observed using a fluorescence microscope.

### ROS detection

HCC cells were passaged into 12-well plates at 2 × 10^5^/well. After 24 h, 10 μM PD2 was added to each well. After 48 h, the cells were stained with a DHR123 staining solution (Sigma, USA). After 30 min of staining, cells were washed once and transferred to flow tubes for detection and analysis with a flow cytometer (CytoFLEX A00-1-1102, USA).

### Transcriptomic analysis

HCCLM3 cells were seeded in 6-well plates 12 h before experiments, then treated with Lico A (30 μM) for 48 h. The cells were collected for transcriptomic analysis. Total RNA extraction was performed on cells treated with Lico A. The extracted total RNA was then subjected to a sample quality check, and samples passing the check were subjected to PCR to obtain cDNA libraries. RNA concentration and purity was measured using NanoDrop 2000 (Thermo Fisher Scientific, Wilmington, DE). RNA integrity was assessed using the RNA Nano 6000 Assay Kit of the Agilent Bioanalyzer 2100 system (Agilent Technologies, CA, USA). The concentration is ≥ 25 (ng/μL), while the OD260/280 is 1.7–2.5 and the OD260/230 is 0.5–2.5. Quality-checked libraries were sequenced in PE150 mode with the Illumina NovaSeq6000 sequencing platform. Transcriptomic analysis was performed using the assay analysis service provided by Biomarker Technologies, Inc.

### Western blotting

The cells were passaged to six-well plates at 2 × 10^5^ cells/well, and cultured in 5% CO_2_ and 37 °C in a cell incubator for 24 h. Next, 1 mL of double-free DMEM was added, followed by PBS, 10 μM PD2. After 48 h, the cells were collected and centrifuged to remove the supernatant, and total cell protein was extracted using a protein extraction kit (Cat# DE101-01, TransGen Biotech, China). Subsequently, the protein concentration was determined using a BCA protein assay kit (P1011, Beyotime Biotechnology, China), and the absorbance was read using a microplate reader. A standard curve was constructed, and the protein concentration of each sample was calculated. The sample was quantified with the minimum protein sample mass. Then, the sample protein was fully denatured, and 30 μg of total protein from each sample was loaded onto SDS-PAGE and transferred onto PVDF membranes. After 2 h of incubation in the blocking solution, the primary antibodies (1:1000) were incubated overnight at 4 °C. The membranes were then incubated with goat anti-rabbit or goat anti-mouse secondary antibodies for 40 min and washed three times with TBST for 10 min each. ECL immunoblotting chemiluminescence solution was used for dark color development. An ultrasensitive multifunctional imager was used for exposure (BIO-RAD ChemiDoc Imaging System 733BR3967, USA) (Additional file 1).

### Co-localization observation assay

Cells in the logarithmic growth phase were seeded at 1 × 10^5^ cells/well in a 12-well cell culture plate pre-coated with cell slides and cultured at 37 °C and 5% CO2. Next, cells were transfected with the plasmid EGFP-LC3 for 24 h. Subsequently, adding 10 μM of PD2 for 48 h, samples were fixed with 4% paraformaldehyde for 30 min. After washing, the membranes were blocked with a blocking solution for 30 min. After discarding the blocking solution, the primary antibody solution was added to the 12-well plate, and the plate was gently shaken at 4 °C overnight. The primary antibody solution was removed, and cells were washed with PBS. Subsequently, the corresponding secondary antibody solution was added to meet different immunofluorescence co-localization requirements. After cleaning with PBS, the climbing piece was removed and attached to a slide. A nail polish seal was used to avoid drying, and photographs were taken under an inverted fluorescence microscope. Analysis of fluorescence intensity using ZEN 2.3 software.

### β-galactosidase staining

HCC cells were passaged into 12-well plates at 2 × 10^5^/ well, and after 24 h, 10 μM PD2 was added to HCC cells. After 48 h, the cells were fixed for 15 min and then stained with a senescence-associated β-galactosidase staining kit (Beyotime, China). The cells were incubated at 37 ℃ overnight and observed using a microscope.

### In vivo anti-tumor assays

SPF female BALB/c nude mice were housed in a sterile environment. SPF sterile mouse food and autoclaved water were fed for one week of adaptive feeding.

HCCLM3 cells in the mid-log phase were selected and digested with 0.25% trypsin. After centrifugation at 500 g for 10 min, cells were washed with PBS, mixed with double-free culture medium, and counted. Then, cells were adjusted to 5 × 10^7^ cells/mL concentration. A 100 μL cell suspension was injected into the right limb of each nude mouse. Seven-ten days after tumor implantation, mice were randomly divided into seven groups: PD2 (5, and 10 mg/kg), 5-FU (10 mg/kg, as positive control), siNIX + 10 mg/kg PD2, siP21 + 10 mg/kg PD2, cyclin A2 + 10 mg/kg PD2, and PBS (control). The drug was administered via intratumoral injection once every three days for four consecutive doses. Body weight change and tumor size of nude mice were recorded. Finally, histopathological tests were performed.

### Statistical analysis

Data are expressed as mean ± standard error. GraphPad Prism software (version 8.0) was used for statistical analysis. T-test and one-way analysis of variance (ANOVA) were used for comparing groups. When P < 0.05 in ANOVA, the Student–Newman–KeuLs test was used for multiple comparisons. P < 0.05 was considered statistically significant.

## Result

### PD2 specifically inhibited the proliferation of HCC cells

**Fig. 1:**
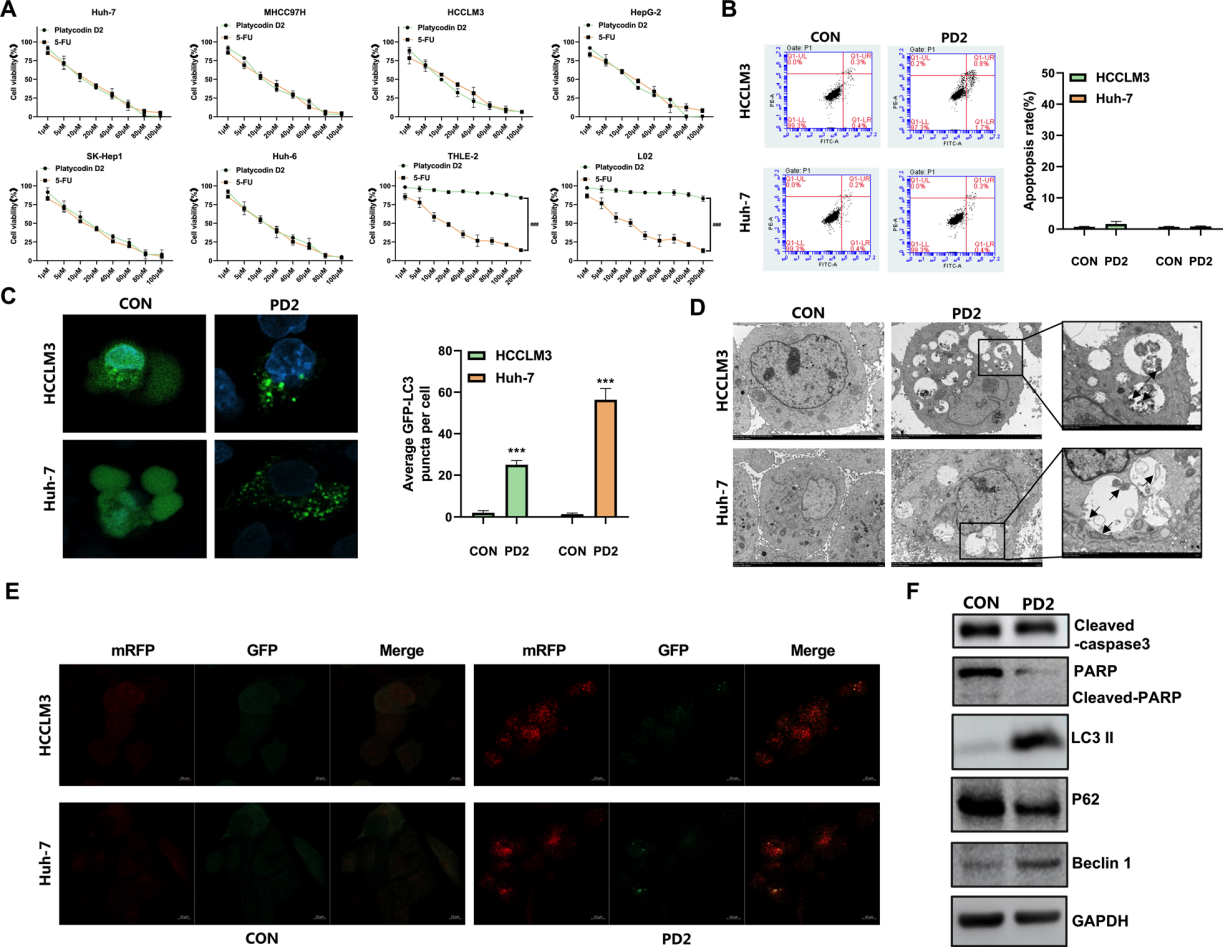
Effect and pathway of PD2 in inhibiting HCC cells **A** CCK-8 assay was used to analyze the inhibitory effect of PD2 on HCC cells and normal liver cells; **B** Flow cytometry for measuring the effects of PD2 on the apoptosis of HCC cells; **C** pGFP-LC3 plasmid was used to analyze whether PD2 induced autophagy in HCC cells; **D** Autophagosome formation was observed by electron microscope; **E** HCC cells were infected with mRFP-GFP-LC3 autophagic double-labeled adenovirus to analyze the formation of autophagolysosome in HCC cells; **F** Western blotting was used to analyze the changes in the expression of apoptosis and autophagy-related proteins in HCCLM3 cells. The scale bar equals 10 µm. ###P < 0.001; Compared with the control group, ***P < 0.001

We analyzed the inhibitory effect of PD2 on HCC cells. First, we used various HCC cell lines to conduct cell activity experiments. We found that similar to the positive drug 5-FU, PD2 significantly decreased the viability of liver cancer cells; however, unlike 5-FU, PD2 had no obvious inhibitory effect on normal liver cells (Fig. 1A and Table 1). The IC_50_ of PD2 on HCC cells was nearly 12 μmol/L, and the IC_50_ value was the smallest for Huh-7 and HCCLM3 cells. These results indicate that PD2 has the potential to be an anti-HCC drug. Based on the IC50 results, we selected 5 and 10 μM doses to treat HCC cells and performed subsequent experiments.

**Table 1:** The inhibitory rate of PD2 on HCC cells

	IC_50_(μmol/L)							
	HCC cells						Normal liver cells	
	Huh-7	MHCC97H	HCCLM3	HepG-2	SK-Hep1	Huh-6	THLE-2	L02
Platycodin D2	10.2 ± 0.3	11.5 ± 0.2	10.4 ± 0.1	11.2 ± 0.3	12.7 ± 0.3	11.8 ± 0.3	> 200	> 200
5-FU	16.8 ± 0.2	16.3 ± 0.3	16.4 ± 0.4	17.3 ± 0.3	16.7 ± 0.3	17.1 ± 0.2	19.2 ± 0.4	18.9 ± 0.4

### PD2 induced autophagy in HCC cells but did not affect their apoptosis

In general, drugs can induce tumor cell death through different types of programmed cell death modes, including apoptosis and autophagy. To verify the pathway by which PD2 induces HCC cell death, we first performed Annexin V flow cytometry and LC3 staining. The results showed that PD2 did not significantly induce apoptosis in HCC cells (Fig. 1B), but significantly induced their autophagy (Fig. 1C). Subsequently, we further analyzed autophagy, and we observed a large number of autophagolysosomes formed by electron microscopy (Fig. 1D); we also found that the number of red fluorescence was significantly higher than the number of green fluorescence after staining the cells with autophagic double-labeled adenovirus, which indicated that the intensity of autophagic flux was increased (Fig. 1E). Finally, apoptosis and autophagy-related proteins were analyzed, and it was found that apoptosis-related proteins had no significant changes, while autophagy-related proteins had significant changes (Fig. 1F). The above results indicated that PD2 was able to induce strong autophagy in HCC cells.

### PD2-mediated autophagy was associated with the removal of damaged mitochondria

**Fig. 2:**
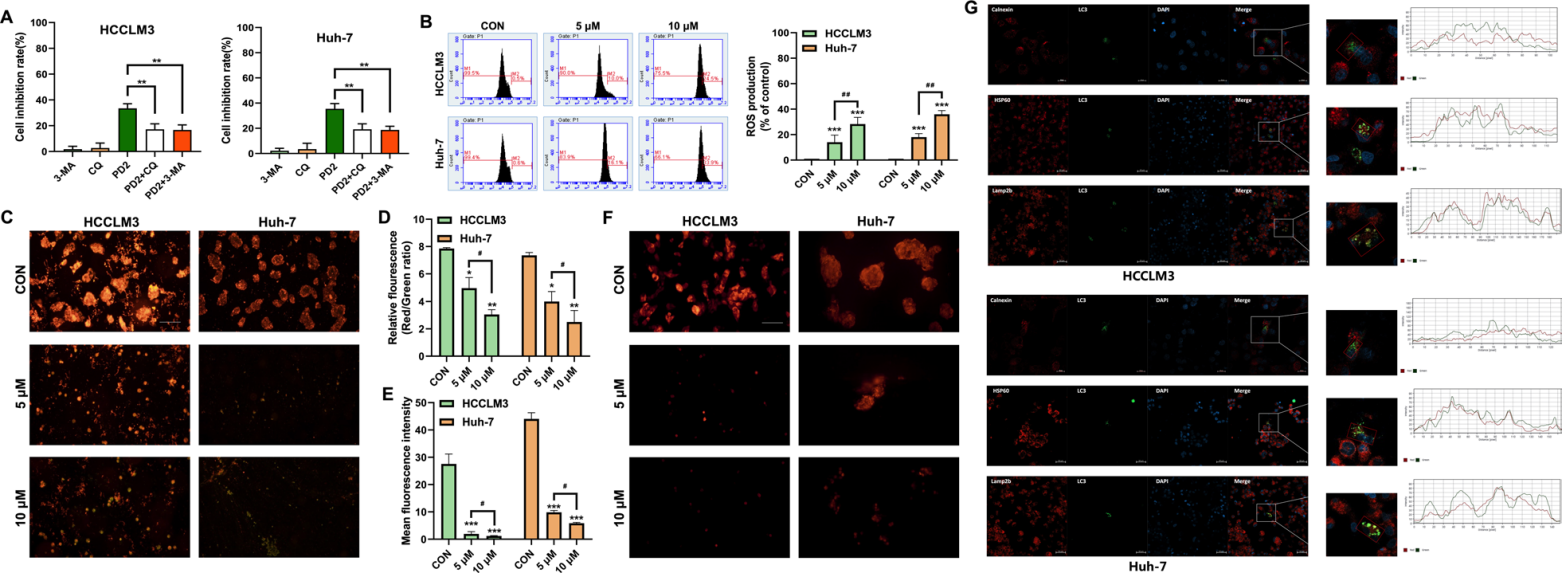
The role of autophagy in the inhibitory effect of PD2 on HCC cell proliferation and mitochondrial damage **A** The inhibitory effect of PD2 on HCCLM3 and Huh-7 cells was analyzed after inhibition of autophagy; **B** ROS level was assessed by flow cytometry. **C** JC-1 staining was used to qualitatively measure mitochondrial polarization; **D** Quantitative analysis of mitochondrial polarization by JC-1 staining; **E**–**F** TMRM staining was used to measure mitochondrial membrane potential. **G** Immunofluorescence assay was used to analyze the colocalization of LC3 with surface proteins of endoplasmic reticulum (Calnexin), mitochondria (HSP60), and lysosomes (Lamp2b) in HCCLM3 and Huh-7 cells. The scale bar equals 10 µm. Compared with the control group, ***P < 0.001, **P < 0.01 and *P < 0.05; For comparison between groups with different concentrations of PD2, ##P < 0.01, and #P < 0.05

To further analyze the role of autophagy in the inhibitory effect of PD2 on HCC cell proliferation, the CCK-8 assay was performed using two autophagy inhibitors. We found that cell death was significantly alleviated after inhibiting autophagy (Fig. 2A). Electron microscopy showed that autophagolysosomes contained damaged mitochondria and endoplasmic reticulum (Fig. 1D). We also analyzed ROS levels and mitochondrial membrane potential, and found that PD2 treatment significantly increased ROS release (Fig. 2B) and reduced mitochondrial membrane potential (Fig. 2C–F). We then assessed the colocalization of LC3 with endoplasmic reticulum, mitochondria, and lysosomes, and found that LC3 co-localized with mitochondria and lysosomes (Fig. 2G). Therefore, we concluded that autophagy induced by PD2 was closely related to the removal of damaged mitochondria.

### PD2 mediated mitophagy in HCC cells through NIX

**Fig. 3:**
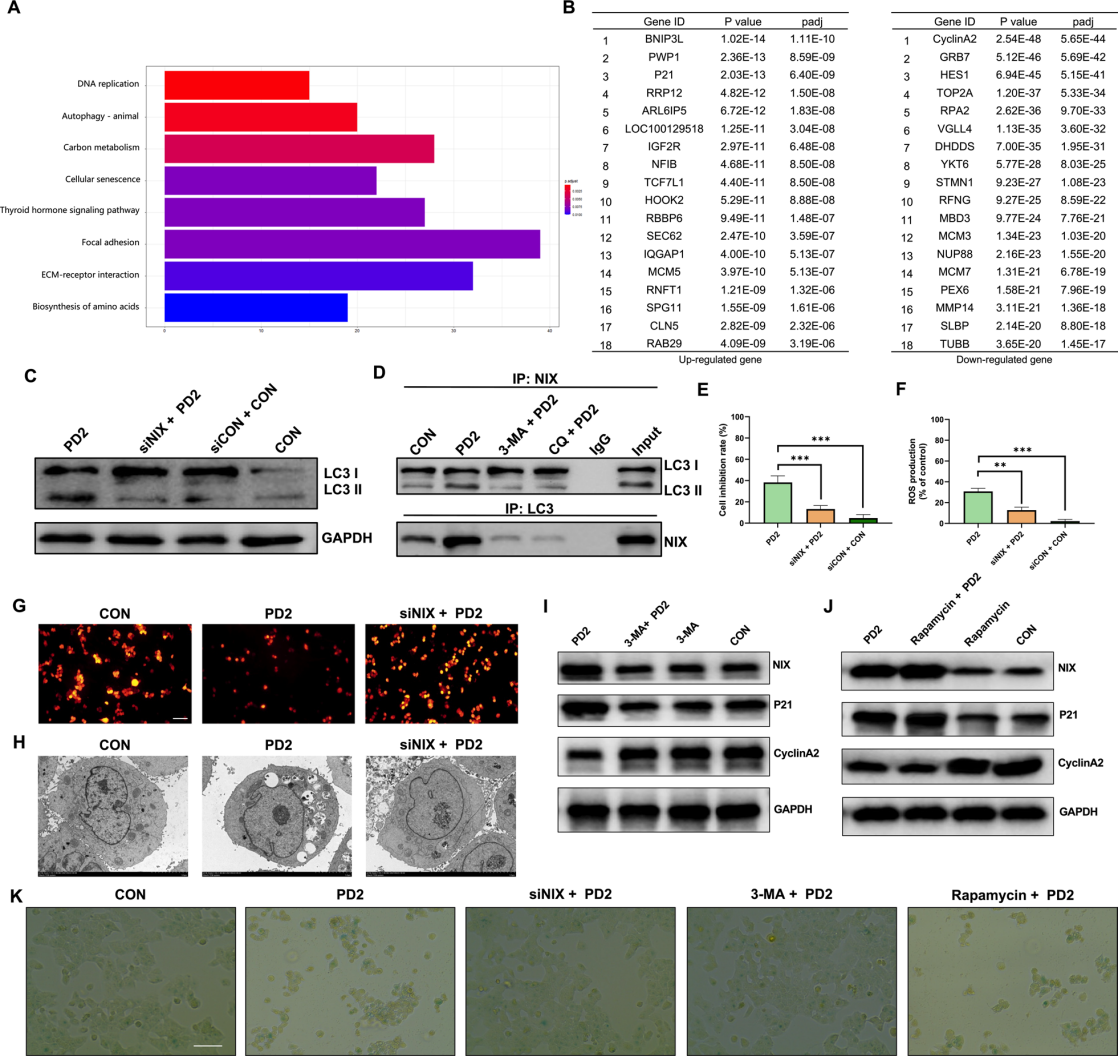
Analysis of the key genes causing HCC cell death. **A** The pathways with significant changes after treating HCCLM3 cells with PD2; **B** Sequencing of significantly upregulated and downregulated genes in HCCLM3 cells after PD2 treatment; **C** LC3 expression was measured after silencing BNIP3L/NIX; **D** Co-IP assay was used to analyze the interaction between NIX and LC3. **E** BNIP3L/NIX silencing was used to analyze the inhibitory effect of PD2 on HCCLM3 cells; **F** BNIP3L/NIX silencing was used to analyze the inhibitory effect of PD2 on ROS production in HCCLM3 cells; **G** Mitochondrial membrane potential was assessed by TMRM staining after BNIP3L/NIX silencing. **H** Autolysosome formation after silencing BNIP3L/NIX was measured by electron microscopy. **I** The expression of NIX, P21 and Cyclin A2 after inhibition of autophagy. **J** The expression of NIX, P21 and Cyclin A2 after increasing of autophagy. **K** Cell senescence was measured by β-galactosidase staining after silencing NIX or inhibiting/increasing autophagy. The scale bar equals 50 µm. Compared with the control group, ***P < 0.001, **P < 0.01 and *P < 0.05

To analyze the key genes that induce autophagy, we used RNA-seq technology to analyze the differential expression of cellular transcriptome after PD2 treatment of HCCLM3 cells. The transcriptomics data showed that PD2 activated a variety of cell death-related pathways. Among them, we found that the autophagy pathway and cellular senescence pathway were activated; thus, we first analyzed the expression of genes related to autophagy (Fig. 3A). We found that the expression of NIX/BNIP3L, a mitochondrial autophagy-related protein, was significantly increased (Fig. 3B). In addition, LC3 level was significantly reduced after NIX silencing (Fig. 3C). Subsequently, we performed Co-IP experiments of NIX and LC3, and found that inhibition of autophagy can significantly reduce the interaction between LC3 and NIX (Fig. 3D). CCK-8 assay showed that the inhibitory effect of PD2 on HCC cells significantly decreased after silencing NIX (Fig. 3E). Subsequently, it was found that silencing NIX significantly reduced PD2-induced ROS overproduction (Fig. 3F), increased mitochondrial membrane potential (Fig. 3G), and inhibited the production of autophagosomes in HCCLM3 cells (Fig. 3H). These results show that NIX induces mitophagy in HCC cells.

### PD2 mediated HCC cell senescence through P21-Cyclin A2

**Fig. 4:**
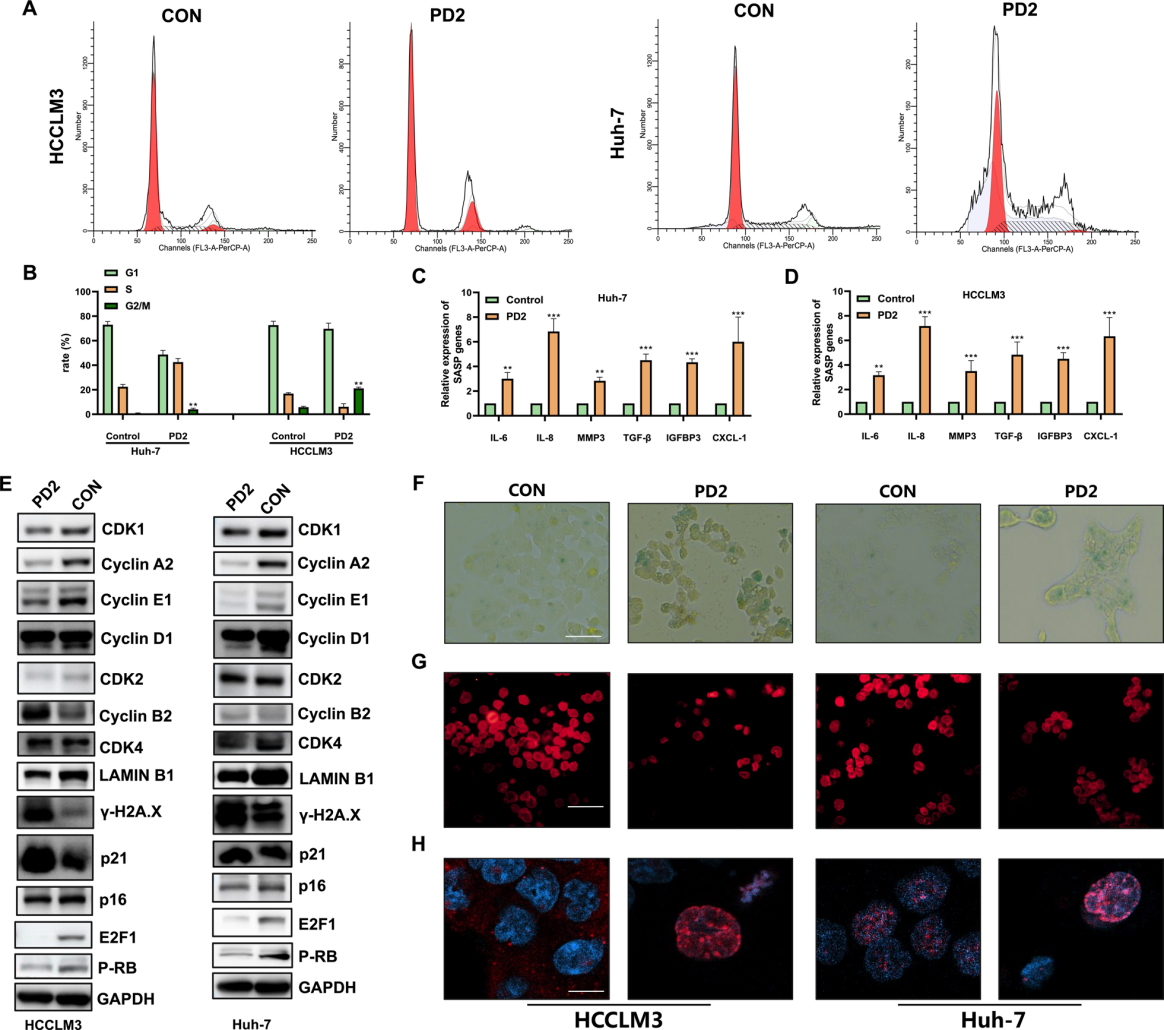
Analysis of cell cycle arrest and cellular senescence. **A**–**B** Flow cytometry was used to analyze the effect of PD2 on the cell cycle of HCC cells; **C**–**D** Quantitative PCR was used to analyze the effect of PD2 on the SASP of HCC cells. **E** Western blotting of cell cycle- and senescence-related proteins; (**F**–**H**) The changes in cell senescence induced by PD2 were analyzed by β-galactosidase staining and immunofluorescence detection of Lamin B1 and γ-H2A.X. The scale bar equals 50 **F**–**G** and 10 **H** µm. Compared with the control group, * * * P < 0.001, **P < 0.01 and *P < 0.05

First, we found that PD2 induced G2/M phase arrest in HCC cells (Fig. 4A–B). Subsequent transcriptomic analysis revealed significant changes in the cellular senescence pathway, evidenced by significant upregulation of P21 and downregulation of CyclinA2 (Fig. 3A–B). Therefore, we measured the expression of cell cycle- and cell senescence-related proteins after PD2 treatment. We found that CDK1, CyclinA2, E2F, and p-RB were significantly downregulated, while P21 and γ-H2A.X were significantly upregulated (Fig. 4E). We also measured the SASP and found that PD2 significantly increased the expression of IL-8, IL-6, MMP3, TGF-β, IGFBP3, and CXCL-1 in HCC cells (Fig. 4C–D). Furthermore, the positive rate of β-galactosidase staining significantly increased (Fig. 4F), the fluorescence intensity of Lamin B1 significantly decreased (Fig. 4G), and γ-H2A.X fluorescence was markedly aggregated in the nucleus after treatment with PD2 (Fig. 4H). These findings indicate that PD2 significantly induced senescence in HCC cells, which may be related to the P21/CyclinA2 signaling pathway.

**Fig. 5:**
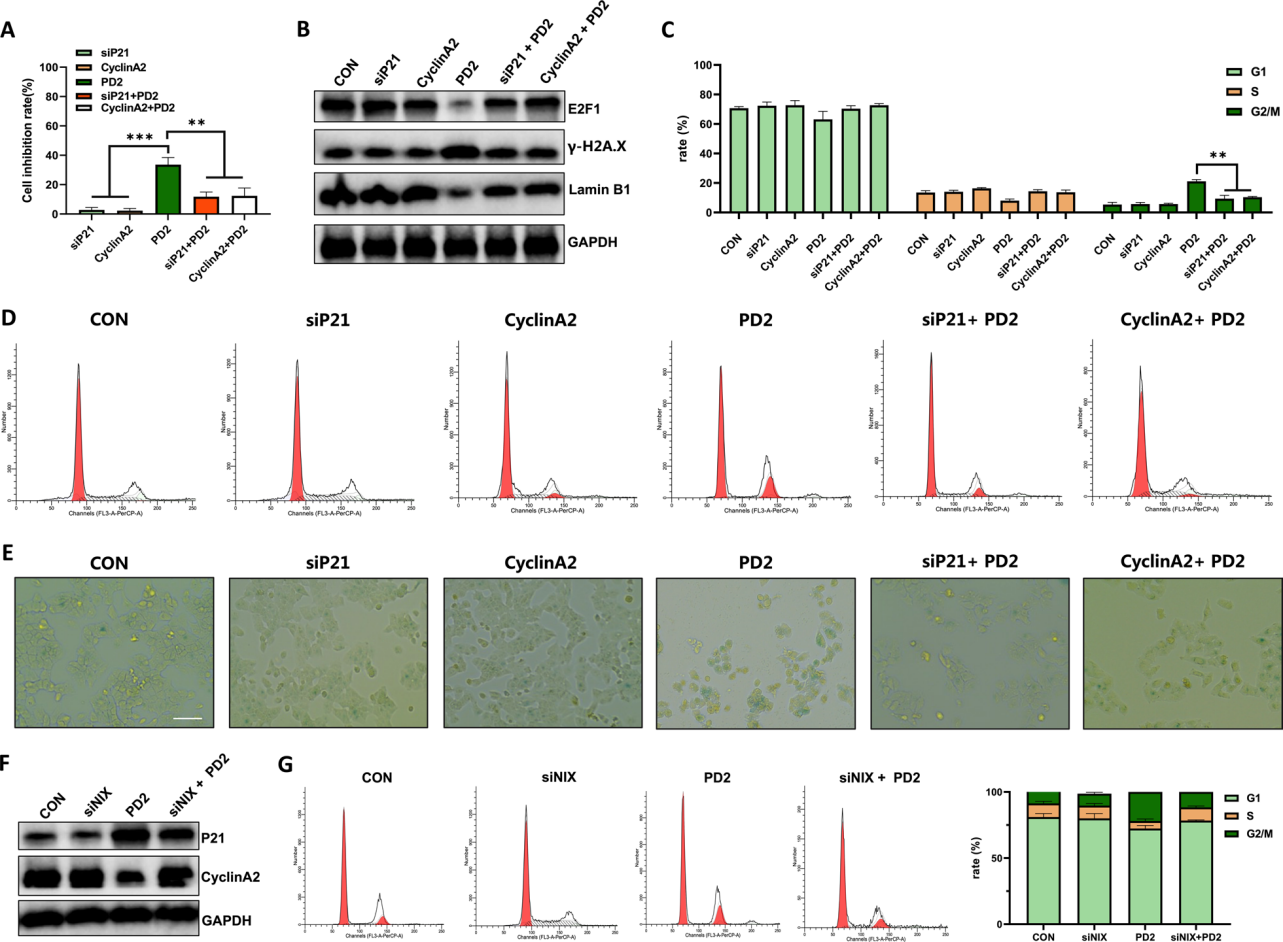
Analysis of key genes causing cell senescence. **A** The inhibitory effect of PD2 on HCCLM3 cells was analyzed after silencing P21 or overexpressing Cyclin A2; **B** After silencing P21 and overexpressing Cyclin A2, the expressions of cell proliferation-related and cell senescence-related proteins were measured; **C**–**D** The changes in cell cycle were measured after P21 silencing and Cyclin A2 overexpression. **E** Cell senescence was measured by β-galactosidase staining after silencing P21 or overexpressing Cyclin A2; **F** The expression of P21 and Cyclin A2 after NIX/BNIP3L silencing; **G** Cell cycle changes were observed after NIX/BNIP3L silencing. The scale bar equals 50 µm. Compared with the control group, * * * P < 0.001, * * P < 0.01 and *P < 0.05

To analyze whether P21 and CyclinA2 are the key regulators of PD2-induced cell senescence in HCC cells, we performed CCK-8 assay, senescence-related protein assay, cell cycle assay, and β-galactosidase staining after silencing P21 and overexpressing CyclinA2, respectively. Both P21 silencing and CyclinA2 overexpression reduced the inhibitory effect of PD2 on HCC cells (Fig. 5A). P21 silencing and CyclinA2 overexpression both reduced the expression of senescence-related proteins (Fig. 5B), alleviated cell cycle arrest at the G2/M phase (Fig. 5C–D) and reduced the number of β-galactosidase-positive cells (Fig. 5E). Our findings showed that PD2 promoted the senescence of HCC cells through the P21/CyclinA2 signaling pathway.

### PD2 induced HCC cell senescence through mitophagy

Based on our results, we found that PD2 induced autophagy and cellular senescence. Therefore, we next analyzed the relationship between autophagy and cell senescence. Recent studies have shown that autophagy both accelerates and inhibits cellular senescence; therefore, we first detected the mitophagy-associated protein NIX and the cellular senescence-associated proteins P21 and cyclinA2 after the addition of autophagy inhibitors and promoters to the treatment of cells with PD2 and found that inhibiting of autophagy was able to reduce the expression levels of the PD2-mediated NIX proteins and the P21 proteins and increase the cyclinA2 expression level (Fig. 3I); while the opposite result was obtained after promoting autophagy (Fig. 3J). A similar trend to the results of the protein level assay was obtained in the β-galactosidase staining assay (Fig. 3K). Subsequently, the changes of cellular senescence-related proteins were analyzed after silencing the autophagy key gene NIX. We found that silencing NIX significantly decreased P21 expression and increased Cyclin A2 expression (Fig. 5F). Then, cell cycle analysis found that silencing NIX significantly alleviated G2/M phase arrest of HCC cells (Fig. 5G), indicating that PD2-mediated mitophagy promoted cellular senescence.

### PD2 induced mitophagy and cellular senescence in vivo

**Fig. 6:**
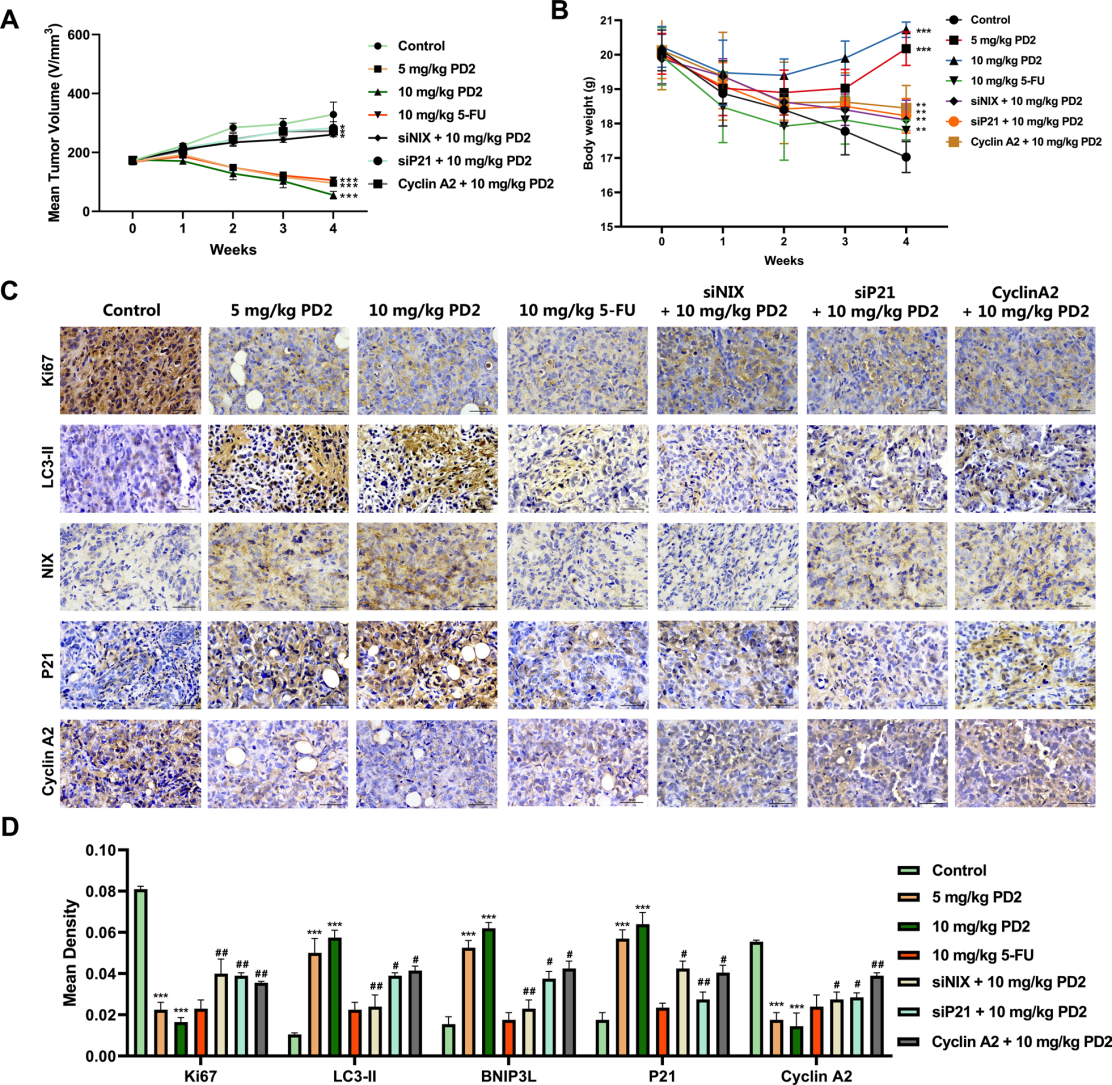
In vivo inhibition of HCC by PD2. Tumor size **A** and mice weight **B** were measured each week. **C**–**D** The expression of Ki67, LC3-II, NIX, P21, and Cyclin A2 in tumor tissues was analyzed by immunohistochemistry. Each group included six mice. The scale bar equals 50 µm. Compared with the control group, ***P < 0.001, **P < 0.01, and *P < 0.05

To verify the anti-tumor effect of PD2 in *vivo*, we used HCC-bearing nude mice. The results indicated that similar to 5-FU, PD2 significantly inhibited tumor growth with a better safety profile (Fig. 6A–B). The body weight of mice in the PD2 treatment group was always higher than that in the model group and 5-FU group (Fig. 6B). Immunohistochemistry showed that PD2 increased the expression of LC3, NIX, and P21, and decreased the expression of Cyclin A2 and E2F1 in tumoral tissues (Fig. 6C–D). These results indicate that PD2 induced mitophagy and cell senescence and inhibited tumor growth in *vivo*.

## Discussion

HCC is a highly aggressive cancer with unsatisfactory outcomes. Currently, surgery and chemotherapy are the main treatment methods for HCC. Although chemotherapy can alleviate patient's condition, most patients relapse shortly after chemotherapy. Therefore, it is urgent to develop new strategies for HCC chemotherapy. Recent studies have shown that aging, a genetic program that is activated by stress, can prevent cell cycle progression and proliferation. Inducing cell senescence is an effective anti-tumor mechanism [8]. At the same time, a large number of studies showed that autophagy is related to inducing cell senescence. Therefore, it is an effective strategy to treat HCC by stimulating autophagy-mediated cell senescence.

This study confirmed that PD2 inhibits HCC growth, has no significant cytotoxic effect on normal hepatocytes, and can induce cell cycle arrest and senescence in HCC cells. In addition, we found that PD2 induced autophagy in HCC cells, leading to mitochondrial damage and ROS release.

Autophagy is a conserved catabolic process. A double-capsule-structured autophagosome forms during autophagy, whose contents are subsequently degraded. Autophagosome encompasses different substrates and can selectively remove different organelles, such as mitochondria. Studies have shown that PARKIN, NIX/BNIP3L, FUNDC1, and BNIP3 play an important role in the early stage of mitophagy [30]. These adaptor proteins have a common core sequence (W/YxxL/I), which can directly interact with Atg8/LC3 or other proteins in this family to mediate mitophagy. NIX is a dual protein that can induce both apoptosis and autophagy. Nix-induced autophagy plays a protective role in some tumor cells; however, NIX-induced autophagy is associated with cell death in other tumor cells. As a BCL2 family protein, NIX competitively binds to BCL2 and disrupts the BCL2-BECN1 complex, releasing BECN1 and promoting autophagosome formation [31]. Similar to NIX, BH3 domain-like small molecules can activate autophagy which can be new targets for drug screening [32]. In this study, it was found that PD2 can enhance autophagy in HCC cells, and RNA-seq technology revealed that PD2 can significantly increase the expression of NIX. The expression of NIX and its binding ability to LC3 were also confirmed by Western blotting and Co-IP assay. Our results indicated that PD2 may mediate autophagy in HCC cells through NIX.

Cell senescence is an irreversible state of cell cycle arrest. P53-P21 and P16 signaling pathways are abnormally activated during cell senescence [33]. Some studies have found that P16 can inhibit CDK4/6 complex and Cyclin D, thereby inducing S phase arrest. P21 can inhibit CDK1 and cyclin A2/B1 complex, resulting in cell cycle arrest at the G2 phase. On the other hand, P21 can inhibit CDK2 and Cyclin A2 complex and induce cell cycle arrest at the S phase [8]. In this study, we found that PD2 significantly activated β-galactosidase in HCC cells and led to cell cycle arrest at the G2 phase. RNA-seq analysis showed that PD2 significantly increased the expression of P21 and reduced the expression of cyclin A2. Western blotting also confirmed the expression of P21 and Cyclin A2, and indicated that P16, CDK2, CDK4, and Cyclin D1 were not significantly downregulated. These results indicate that PD2 may induce senescence of HCC cells by modulating P21/Cyclin A2 signaling pathway. Subsequently, silencing and overexpression of P21 and Cyclin A2 showed that silencing P21 or overexpression of Cyclin A2 alleviated PD2-mediated senescence of HCC cells, indicating that P21/Cyclin A2 signaling pathway is a key pathway for PD2-mediated senescence of HCC cells.

Previous studies have shown that autophagy can induce senescence [8, 34]. Several studies have shown that autophagy plays a positive or negative role in the induction and maintenance of senescence. Studies have found that impaired autophagy can aggravate DNA damage, which eventually causes permanent cell cycle arrest or necrosis [8]. Moreover, various anticancer agents induce excessive autophagy, which affects the dynamic stability of Cyclins and leads to cell cycle arrest, senescence, and autophagy-related cell death [35]. This study showed that PD2 induced both autophagy and cell senescence, indicating its great potential as an anti-HCC drug. Subsequently, through transcriptomic analysis and protein level detection, it was found that PD2 induced mitophagy through NIX, which in turn mediated cell senescence through P21/Cyclin A2 signaling pathway.

Previously, the anti-tumor properties of PD2 have been rarely studied, and the molecular mechanisms behind the cytotoxic effects of PD2 have not been precisely elucidated. This study demonstrated that PD2 promotes NIX-induced mitophagy and P21/Cyclin A2-mediated cell senescence of HCC cells in vivo and in vitro. The results of this study can improve the treatment of HCC and provide an experimental and theoretical basis for the development of PD2-based anti-tumor drugs in the future.

### Supplementary Information

**Additional file 1: Figure S1.** Western blot identified the efficacy of siNIX, siP21 silencing, and CyclinA2 overexpression. **Figure S2.** Western blot identified the expression of NIX in HCC cells and normal hepatocytes.

## Data Availability

The datasets used and analyzed during the current study are available from the corresponding author on reasonable request.
