# Supplementary material for: Platycodin D2 enhances P21/CyclinA2-mediated senescence of HCC cells by regulating NIX-induced mitophagy
Source: Cancer Cell Int. 2024 Feb 19;24:79. doi: 10.1186/s12935-024-03263-y (PMC10875888; doi:10.1186/s12935-024-03263-y)
Supplement: Supplementary file 1 — Additional file 1: Figure S1. Western blot identified the efficacy of siNIX, siP21 silencing, and CyclinA2 overexpression. Figure S2. Western blot identified the expression of NIX in HCC cells and normal hepatocytes. [file 12935_2024_3263_MOESM1_ESM.pdf]

Supplementary information

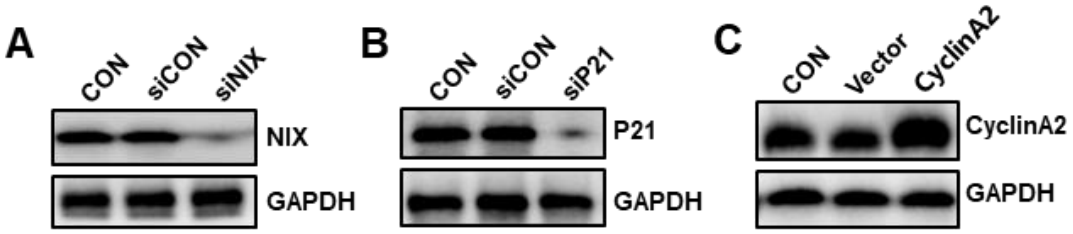

Figure S1. Western blot identified the efficacy of siNIX, siP21 silencing, and CyclinA2 overexpression.

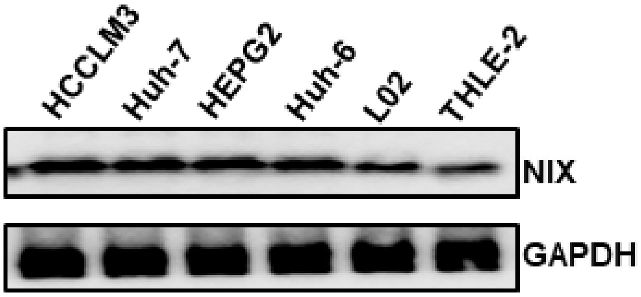

Figure S2. Western blot identified the expression of NIX in HCC cells and normal hepatocytes.
